# Telehealth Adoption and Discontinuation by US Hospitals: Results From 2 Quasi-Natural Experiments

**DOI:** 10.2196/28979

**Published:** 2022-02-18

**Authors:** Aaron Baird, Yichen Cheng, Yusen Xia

**Affiliations:** 1 Institute of Health Administration Georgia State University Atlanta, GA United States; 2 Department of Computer Information Systems Robinson College of Business Georgia State University Atlanta, GA United States; 3 Institute for Insight Robinson College of Business Georgia State University Atlanta, GA United States

**Keywords:** telehealth, hospitals, adoption, discontinuation, health information system

## Abstract

**Background:**

Prior US hospital telehealth (video visit) studies have focused on describing factors that influence telehealth adoption or performance effects for specific patient segments, hospital systems, or geographic regions. To our knowledge, a larger-scale, national-level (US) study has yet to be conducted on the causal impacts of hospital telehealth adoption as well as discontinuation.

**Objective:**

The aim of this study is to understand the causal impact of US hospital telehealth adoption or discontinuation on hospital performance from 2016 to 2018.

**Methods:**

We analyzed impacts of telehealth adoption or discontinuation by US hospitals on emergency department visits, total ambulatory visits (minus emergency department visits), outpatient services revenue, total facility expenses, and total hospital revenue for the 2016-2018 period. We specifically focused on performance effects for hospitals that switched from not having telehealth to adopting telehealth, or vice versa, during the 2016-2018 period, thus exploiting 2 quasi-natural experiments. We applied a difference-in-differences research design to each of the 2 main analyses. We compared hospitals that have made a telehealth change to groups of hospitals with similar characteristics that did not make a telehealth change, which established a counterfactual. To appropriately match hospitals between treatment and control groups, we applied propensity score matching. Our primary data were from the American Hospital Association Annual Survey and the Healthcare Cost Report Information System data. Several control variables were obtained from additional sources, including the Area Health Resource File and the Federal Communications Commission.

**Results:**

We found that telehealth adoption by US hospitals during the 2016-2018 period resulted in, on average, an increased number of total ambulatory visits (*P*=.008), increased total facility expenses (*P*<.001), and increased hospital revenue (*P*=.004) compared with the control group. We found that telehealth discontinuation during the same period resulted in, on average, decreased outpatient services revenue (*P*=.02) compared with the control group.

**Conclusions:**

Our findings suggest that telehealth adoption increases use but has mixed impacts on performance, given that cost and revenue increase. However, once telehealth is offered, removing it can have a negative impact on performance, implying that returning to prior performance levels, if telehealth is removed, may be challenging.

## Introduction

### Background

Telehealth, in the form of video visits between health care providers and patients (*telehealth*, henceforth), is used by hospitals and their affiliated clinics to maintain or improve access to postdischarge follow-up, continuity of care, and care for nonurgent issues [1-5]. Although a number of studies have evaluated the impacts of telehealth on outcomes [2,6-10], such studies have primarily focused on either the determinants of telehealth adoption [11] or effects of telehealth primarily for patient populations limited to specific hospital systems or regions [4,12-14]. Larger-scale studies exploiting national-level natural variation in telehealth adoption as well as discontinuation over multiple years have yet to be conducted.

Overall, although many view telehealth with optimism, we do not yet fully understand the impact on hospital-level outcomes when telehealth is adopted or, in the case of challenges, discontinued. Thus, this study seeks to understand such impacts, including impacts resulting from telehealth discontinuation, which is not an aspect of telehealth that has been considered yet in the literature. For instance, in regard to challenges that may lead to discontinuation of telehealth, it is well known that telehealth can be especially difficult to sustain and integrate with workflows designed for in-person interactions [7] and can result in variable outcomes [15,16]. Particular challenges for hospitals offering telehealth include prioritization of the success of telehealth; engagement by providers, patients, and leaders; and continuous improvement [17]. Many times, telehealth is initially viewed with optimism, but the reality is that many clinicians have stopped using it in the past after a few visits [17]. Especially important to mitigate such issues are deliberate efforts to create protocols, develop appropriate scheduling techniques, and formalize an understanding for when telehealth is and is not appropriate [18], which, if not addressed, can lead to significant challenges, resistance, or program failure. Furthermore, the effects of telehealth have been found to have mixed or even positive effects on costs [3,19]. In the case of telehealth substituting for expensive in-person visits such as visits to the emergency department (ED) or in-patient admissions, telehealth can be cost-effective [20,21]. However, when offering video-based consultations to patients, it is also possible that increased access to health care increases provider costs and the number of visits requested by patients, which can result in less revenue, especially if telehealth is reimbursed at a lower rate than in-person visits [3].

Finally, telehealth is a particularly interesting case because it can be technically relatively easy to adopt or discontinue, especially if using a vendor-supported or cloud-based system, but, as discussed previously, can simultaneously result in significant and costly workflow challenges [8,22]. It is well known that telehealth use is an excellent opportunity to enhance access to care, but it is also well known that inadequate barrier identification and management can doom telehealth pilots [17,23]. Furthermore, given the variety of factors that may influence telehealth adoption, use, and potential discontinuation, several factors, including hospital and regional characteristics, must be controlled for. Thus, this study comprehensively examines both telehealth adoption and discontinuation in the United States from 2016 to 2018 through analysis of 2 quasi-natural experiments (ie, one for adoption and one for discontinuation), while controlling for several potential confounding variables. We also conduct robustness checks to validate our findings.

### Implications

Our primary findings are as follows: (1) telehealth adoption by US hospitals during the period studied resulted in increased ambulatory visits, increased facility expenses, and increased hospital revenue in comparison with the control group, and (2) telehealth discontinuation resulted in decreased outpatient services revenue in comparison with the control group. The implications are that adopting telehealth increases use of ambulatory services, which implies greater access, but these findings also suggest that profit performance will likely be mixed. Furthermore, removing telehealth once offered can negatively affect future performance, implying that performance levels likely will not simply return to what they were before telehealth was adopted and then subsequently discontinued. Further implications are discussed later.

## Methods

### Overview

To address our research objectives, we analyzed the impact of telehealth adoption or discontinuation by US hospitals from 2016 to 2018 using difference-in-differences estimation of 2 quasi-natural experiments: (1) US hospital telehealth *adoption* during the period considered and (2) US hospital telehealth *discontinuation* during the same period. We specifically considered impacts of telehealth adoption or discontinuation during this period on ED visits, total ambulatory visits (minus ED visits), outpatient services revenue, total facility expenses, and hospital revenue (a more detailed description of these dependent variables is available in Multimedia Appendix 1).

### Data

Data on which US hospitals continued to offer, or discontinued, telehealth were obtained from the American Hospital Association (AHA) Annual Survey for 2016-2018 (although data quality may be a concern, prior studies such as the one by Adler-Milstein et al [11] have found the AHA data to be highly consistent with the data from the Healthcare Information and Management Systems Society data set, suggesting high data quality). Outcome data for ED visits, total ambulatory visits, outpatient services revenue, total facility expenses, and hospital revenue per US hospital were obtained from the 2016-2018 AHA Annual Survey and the AHA’s Centers for Medicare & Medicaid Services Healthcare Cost Report Information System (HCRIS) data (ie, AHA’s version of the Centers for Medicare & Medicaid Services HCRIS data). Covariates used for propensity score matching and controls were obtained from the AHA data sets and from the US county-level data available from the Area Health Resource File, as well as the Area Deprivation Index (ADI) sourced from BroadStreet, health rankings data from the University of Wisconsin Population Health Institute, and supplementary data from the Federal Communications Commission for broadband speeds per county. We included several controls from these data sources to account for rival explanations. Controls and covariates were derived from a literature review [24-30]. Tables 1 and 2 describe the relevant variables.

**Table 1:** Telehealth adoption sample descriptive statistics averaged for 2016-2018 (for 135 US hospitals that did not have telehealth for all 3 years or started to adopt telehealth in 2017 or 2018).

Group and variable		Description	Value, N	Values, mean (SD; range)
**Telehealth adoption and outcomes**				
	Telehealth video-based consultation	Whether a hospital adopted telehealth in a given year	405	0.39 (0.49; 0-1)
	EDVisits^a^ (in thousands)	Number of emergency department visits	405	47.02 (32.98; 0-174.96)
	TotAmbVisits^a^ (in thousands)	Total number of ambulatory visits (minus emergency department visits)	405	196 (209.36; 2.09-1488.13)
	OutpatSerRev^a^ (in millions, US $)	Outpatient services revenue	393	179 (312.35; 0-2831.15)
	TotFacExp^a^ (in millions, US $)	Total facility expenses	405	321.61 (342.3; 18.97-2687.47)
	HospRev^a^ (in millions, US $)	Total hospital revenue	393	152.34 (283.84; 0-2399.62)
**Hospital-level variables**				
	SystemOwned	System ownership	405	0.71 (0.45; 0-1)
	WageIndx	Index of hospital labor market wages	393	1.01 (0.15; 0.72-1.35)
	HITAssetCost (in millions, US $)	Health information technology asset acquisition costs	393	0.94 (3.81; 0-29.8)
	TotAdmAndVsts (in thousands)	Sum of inpatient admissions and outpatient visits	405	303.62 (276.89; 5.19-1853.46)
	Herfindahl-Hirschman Index	Competition index (1=monopoly) per hospital referral region	405	0.13 (0.09; 0.03-0.56)
	PercMdcdElig	Percentage Medicaid eligibility	405	0.22 (0.08; 0.07-0.5)
	COTH	Teaching hospital	405	0.09 (0.29; 0-1)
	Own_FP	For-profit ownership	405	0.12 (0.32; 0-1)
	Own_NP	Not-for-profit ownership	405	0.77 (0.42; 0-1)
	Own_Gov	Government ownership	405	0.11 (0.31; 0-1)
	PercCapit	Percentage of net patient revenue capitated	375	0.79 (4.17; 0-53)
	PercRsk	Percentage of net patient revenue shared risk	347	1.77 (5.41; 0-42)
	Region_MW	Midwestern region	405	0.31 (0.46; 0-1)
	Region_S	Southern region	405	0.37 (0.48; 0-1)
	Region_W	Western region	405	0.02 (0.14; 0-1)
	Region_NE	Northeast region	405	0.3 (0.46; 0-1)
	CMI	Case Mix Index	393	1.74 (0.33; 0.93-2.93)
	Urban	1 if urban location	405	1 (0)
**County-level variables**				
	CntyHlthRank	Normalized within state county health rankings for health outcomes, 1 being best	405	0.4 (0.32; 0-1)
	CntyPercPop65	Percentage of population aged >65 years	405	0.15 (0.03; 0.09-0.26)
	CntyPercBlack	Percentage of population Black	405	15.04 (11.97; 0.6-63.7)
	CntyPercNative	Percentage of population Native	405	0.6 (1.53; 0.1-17.5)
	CntyPercLatino	Percentage of population Latino	405	12.89 (11.89; 0.8-60.6)
	CntyPercDpPov	Percentage of population in deep poverty	405	6.64 (2.59; 2.2-16)
	CntyPercDisabled	Percentage of population disabled	405	10.01 (3.05; 5.1-17.2)
	CntyBBMaxUP	Maximum advertised broadband upload speed	405	26.68 (32.56; 1.56-160.98)
	CntyHsholdIntUse	Percentage of households who report using the internet	405	0.87 (0.04; 0.72-0.97)
	CntyADI	Area Deprivation Index (10=most deprived)	405	5.04 (1.51; 1.53-8.4)

^a^More details about the outcome variables are available in Multimedia Appendix 1.

**Table 2:** Telehealth discontinuation sample descriptive statistics averaged for 2016-2018 (for 524 US hospitals that had telehealth for all 3 years or started to remove telehealth in 2017 or 2018).

Group and variable		Description	Value, N	Values, mean (SD; range)
**Telehealth adoption and outcomes**				
	Telehealth video-based consultation	Whether a hospital adopted telehealth in a given year	1572	0.93 (0.26; 0 to 1)
	EDVisits^a^ (in thousands)	Number of emergency department visits	1572	61.16 (49.82; 0 to 617.78)
	TotAmbVisits^a^ (in thousands)	Total number of ambulatory visits (minus emergency department visits)	1572	263.74 (413.69; 1.02 to 6497.28)
	OutpatSerRev^a^ (in millions, US $)	Outpatient services revenue	1550	247.07 (476.36; –1443.98 to 6717.17)
	TotFacExp^a^ (in millions, US $)	Total facility expenses	1572	440.55 (565.66; 13.91 to 6004.75)
	HospRev^a^ (in millions, US $)	Total hospital revenue	1550	188.32 (377.16; 0 to 7055.45)
**Hospital-level variables**				
	SystemOwned	System ownership	1572	0.86 (0.35; 0 to 1)
	WageIndx	Index of hospital labor market wages	1550	0.98 (0.14; 0.71 to 1.44)
	HITAssetCost (in millions, US $)	Health information technology asset acquisition costs	1550	2.97 (12.94; 0 to 175.42)
	TotAdmAndVsts (in thousands)	Sum of inpatient admissions and outpatient visits	1572	407.36 (493.07; 3.82 to 6989.63)
	Herfindahl-Hirschman Index	Competition index (1=monopoly) per hospital referral region	1572	0.15 (0.12; 0.03 to 0.96)
	PercMdcdElig	Percentage Medicaid eligibility	1572	0.21 (0.07; 0.05 to 0.5)
	COTH	Teaching hospital	1572	0.16 (0.37; 0 to 1)
	Own_FP	For-profit ownership	1572	0.11 (0.31; 0 to 1)
	Own_NP	Not-for-profit ownership	1572	0.8 (0.40; 0 to 1)
	Own_Gov	Government ownership	1572	0.09 (0.29; 0 to 1)
	PercCapit	Percentage of net patient revenue capitated	1476	0.53 (2.62; 0 to 40)
	PercRsk	Percentage of net patient revenue shared risk	1381	2.28 (7.33; 0 to 81)
	Region_MW	Midwestern region	1572	0.33 (0.47; 0 to 1)
	Region_S	Southern region	1572	0.42 (0.49; 0 to 1)
	Region_W	Western region	1572	0.03 (0.17; 0 to 1)
	Region_NE	Northeast region	1572	0.21 (0.41; 0 to 1)
	CMI	Case Mix Index	1550	1.68 (0.26; 0.99 to 2.68)
	Urban	1 if urban location	1572	1 (0; 1 to 1)
**County-level variables**				
	CntyHlthRank	Normalized within state county health rankings for health outcomes, 1 being best	1572	0.39 (0.3; 0 to 1)
	CntyPercPop65	Percentage of population aged >65 years	1572	0.15 (0.04; 0.09 to 0.35)
	CntyPercBlack	Percentage of population Black	1572	14.19 (12.71; 0.4 to 69.1)
	CntyPercNative	Percentage of population Native	1572	0.64 (2.38; 0.1 to 38.4)
	CntyPercLatino	Percentage of population Latino	1572	12.2 (13.79; 0.5 to 90.6)
	CntyPercDpPov	Percentage of population in deep poverty	1572	6.6 (2.59; 1.8 to 19.9)
	CntyPercDisabled	Percentage of population disabled	1572	10.2 (2.92; 4.2 to 20.7)
	CntyBBMaxUP	Maximum advertised broadband upload speed	1572	22.02 (26.32; 1.26 to 160.98)
	CntyHsholdIntUse	Percentage of households who report using the internet	1572	0.87 (0.05; 0.6 to 0.97)
	CntyADI	Area Deprivation Index (10=most deprived)	1572	5.31 (1.35; 2.01 to 8.93)

^a^More details about the outcome variables are available in Multimedia Appendix 1.

### Statistical Analyses

We applied difference-in-differences (DID) estimation with propensity score matching at the firm (hospital) unit of analysis to understand the effect of telehealth adoption and discontinuation by US hospitals during the 2016-2018 period. We conducted 2 primary analyses that exploited 2 quasi-natural experiments. The first DID analysis focused on telehealth adoption and evaluated impacts on performance for hospitals that went from no telehealth to offering telehealth during this period. The second DID analysis focused on telehealth discontinuation and evaluated impacts for hospitals that went from offering telehealth to discontinuing telehealth during this period. Control group selection and formation is discussed later in this section. This design followed other notable studies that assessed the impact of health information technology adoption and use on outcomes [28-31] as well as recommendations on effectively estimating causal effects by means of observational data [32,33]. This design is appropriate for estimating causal effects when pre- and posttreatment observational data are available, treatment and control groups with sufficiently balanced covariates and common trends before treatment can be established, and exogenous shocks can be assumed to be consistent between groups [34].

For the telehealth adoption analysis, treatment hospitals are those that first did not offer telehealth but then offered telehealth in a subsequent year. As we have 3 years of data that include the telehealth video visit (yes or no) question, we restricted our focus to video visits for chronic conditions or postsurgical follow-up as opposed to also including consideration of telehealth related to remote patient monitoring and mental health and addiction as separately measured in the AHA Annual Survey. For all US hospitals surveyed by the AHA for this quasi-natural experiment, treatment hospitals are those that (1) did not offer telehealth in 2016 but started in 2017 or 2018 (group 1, n=71) or (2) did not offer telehealth in 2016 or 2017 but then started offering it in 2018 (group 2, n=14). Control hospitals are those that did not offer telehealth in all 3 years (n=50).

For the telehealth discontinuation analysis, treatment hospitals are those that offered telehealth but then discontinued it in a subsequent year. For this quasi-natural experiment, the treatment hospitals are those that (1) offered telehealth in 2016 but discontinued in 2017 or 2018 (group 1, n=12) or (2) offered telehealth in both 2016 and 2017 but discontinued in 2018 (group 2, n=80). Control hospitals are those that offered telehealth in all 3 years (n=432).

To balance the covariates between the treatment and control groups in each of these analyses, we applied propensity scoring and, subsequently, matching. Propensity scoring is applied by first determining the propensity of a hospital being in the treatment group, given observable covariates [35,36]. Then, to reduce selection bias, a matching technique is used to find control group participants (hospitals, in this case) that ultimately result in no observable significant covariate differences between treatment and control groups [35]. Similar to Oh et al [30] and Bao et al [29], we calculated propensity scores by means of logistic regression for each of the analyses (ie, for the adoption analysis and then again for the discontinuation analysis), as explained further in this section. Our covariates consisted of both hospital-level variables and county-level variables, with SEs clustered at the hospital level to account for repeated county-level observations for hospitals within the same county. The logistic regression analysis results for propensity scores are reported in Multimedia Appendix 1.

Using the scores that resulted from obtaining predicted values per hospital, we applied one-to-many matching using both the propensity score and covariates (a one-to-one matching procedure was also tested, as reported in Multimedia Appendix 1, and the results were similar). Matched hospitals belonged to the same teaching, urban, and system status. In addition, we matched hospitals with similar sizes by restricting hospital size (total admissions plus visits) to a difference of no more than a factor of 1.5 and a difference in propensity scores of no more than 0.1. Therefore, for each treatment hospital, we had a cluster of hospitals as the control. For telehealth adoption, the result was a treatment group consisting of 85 hospitals and a matched sample control group consisting of 85 hospital clusters, with an average size of 2 controls and a median size of 1 control in each hospital cluster. For telehealth discontinuation, the result was a treatment group consisting of 92 hospitals and a matched sample control group consisting of 92 hospital clusters, with an average size of 28 controls and a median size of 17 controls in each hospital cluster. We used averaged outcomes (ED visits, total ambulatory visits, outpatient services revenue, total facility expenses, and hospital revenue) for each observed control cluster. The matching for hospitals in group 1 was conducted based on the propensity score and covariates observed at year 2016, and the matching for group 2 was based on observations at year 2017. Comparison of covariates between the 2 groups resulted in no significant differences.

To obtain the propensity score, we conducted a logistic regression analysis using treatment group membership (1 for yes and 0 for no) as the dependent variable for the adoption analysis and then again for the discontinuation analysis. We applied a collection of hospital and county level characteristics as the independent variables for each analysis, with the same control variables being used in each propensity score model. Let *p_it_*=*P*(*hospital i in the treatment group*) with the following formula:


ln(*p_it_*/1–*p_it_*) = *β*_0_ + *β'*_1_*X_it_*

*β*_0_ is the constant and *X_it_* represents factors that affect a hospital’s decision of whether telehealth existed for the adoption analysis (1=hospital is in the treatment group and therefore adopted telehealth in 2017 or 2018) or was discontinued for the discontinuation analysis (1=hospital is in the treatment group and therefore discontinued telehealth in 2017 or 2018). *β'*_1_ is the coefficient vector.

Next, identification of the change in ED visits, total ambulatory visits, outpatient services revenue, total facility expenses, and hospital revenue after telehealth adoption and discontinuation was derived through the following DID model, applied once to the adoption analysis and once to the discontinuation analysis. Note that when conducting the analyses, we combined hospitals from group 1 and group 2 as the treatment group. *β*_0_ is the constant, *β*_1_ is the effect from the treatment group, *β*_2_ represents posttreatment periods, and *β*_3_ is the treatment effect (ie, the DID effect), which is the expected value difference in the time trend as well as the difference between treatment and control groups after treatment. We included hospital fixed effects (*μ_i_*) to address any time-invariant hospital heterogeneity and time fixed effects to address time trends (*ϑ_t_*). We performed an estimation using ordinary least squares [31]. The DID equation representing our model is as follows:

*Y_it_* = *β*_0_ + *β*_1_*T_it_* + *β*_2_*p_it_* + *β*_3_ (*T_it_* × *p_it_*) + *μ_i_* + *ϑ_t_* + *ε_it_*

### Robustness

Threats to validity could include endogeneity of telehealth adoption and decision-making around discontinuation, especially if our sample was subject to selection bias. We addressed this concern by also conducting Heckman analyses. Furthermore, nonrandom market changes, after treatment, may differentially affect outcomes [37]. For instance, perhaps broadband infrastructure or household use of the Internet expanded or contracted at different rates between the control and treatment groups in or after 2017 or 2018. These threats were addressed with our propensity scoring and matching approach that included county-level maximum broadband speeds and household internet use as covariates in the logistic regression analysis, in addition to several other covariates considered when scoring and matching. For instance, we also included the ADI in our propensity score matching procedure to address regional economic states and, potentially, changes over time such as changes after treatment that are not fully addressed in a DID model. Overall, we included several hospital-level (eg, Case Mix Index, hospital size, and market competition) and county-level covariates (eg, maximum broadband speeds, household internet use, county health ranking, and ADI) to address a variety of potential threats to validity (eg, differences in broadband penetration affecting telehealth adoption or outcomes). Finally, we also tested whether outcomes change in the years *after* treatment to provide additional explanatory information.

## Results

### Common Trends

**Figure 1:**
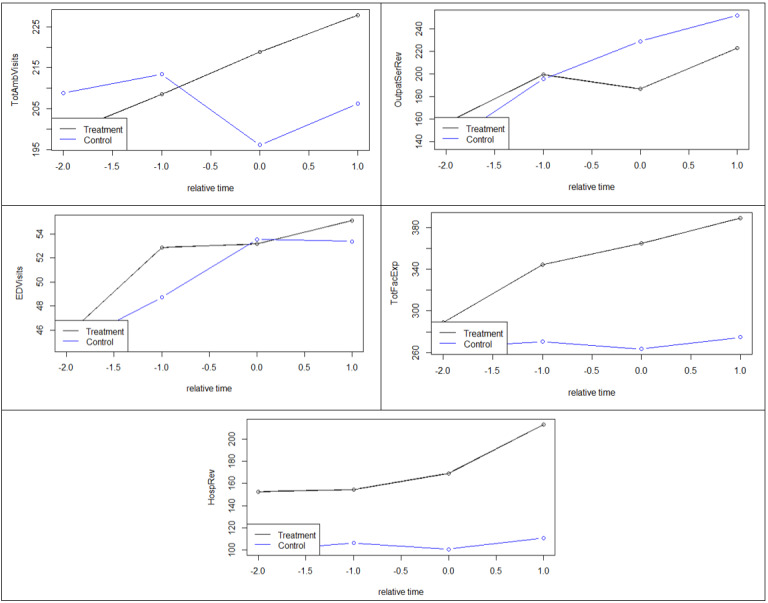
Common trends per outcome per year relative to when telehealth was adopted. EDVisits: emergency department visits; HospRev: hospital revenue; OutpatSerRev: outpatient services revenue; TotAmbVisits: total ambulatory visits; TotFacExp: total facility expenses.

**Figure 2:**
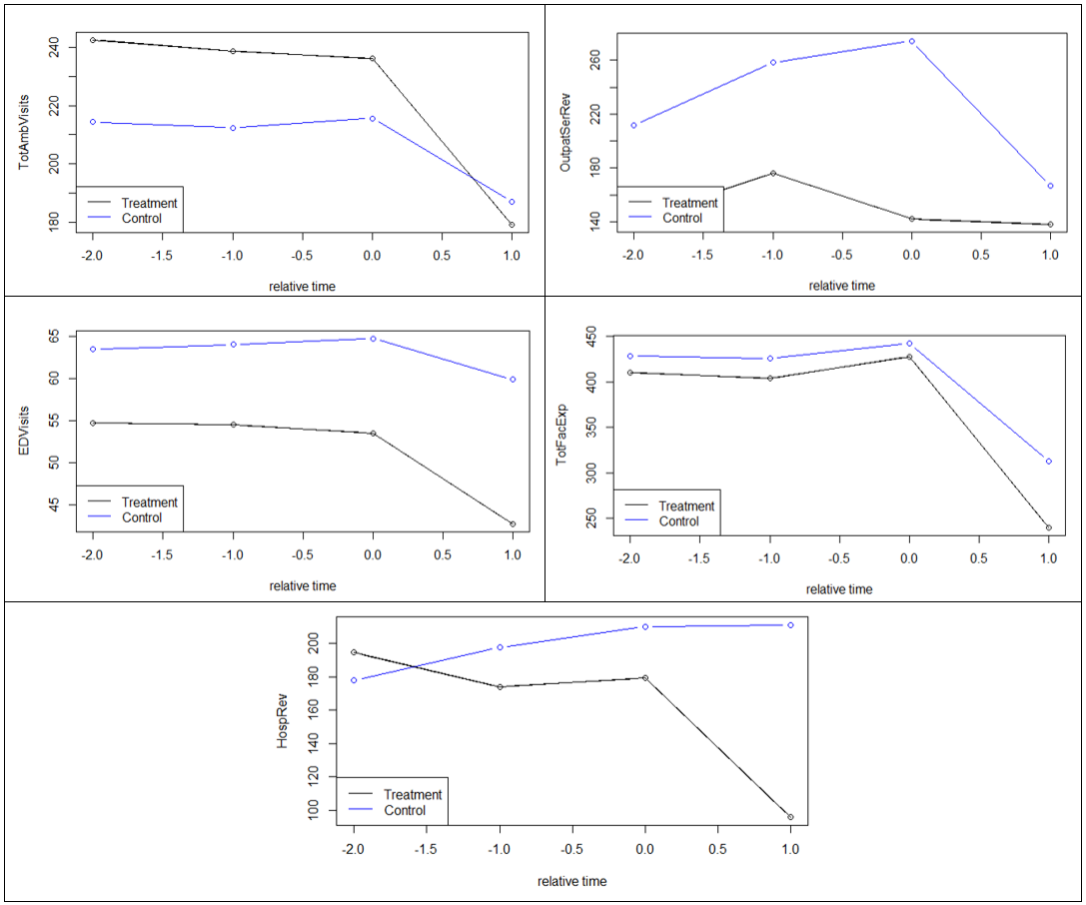
Common trends per outcome per year relative to when telehealth was discontinued. EDVisits: emergency department visits; HospRev: hospital revenue; OutpatSerRev: outpatient services revenue; TotAmbVisits: total ambulatory visits; TotFacExp: total facility expenses.

For testing common trends, we plotted the averages of ED visits, total ambulatory visits, outpatient services revenue, total facility expenses, and hospital revenue for each of the groups at points in time (years) *relative* to when telehealth was adopted (Figure 1) or discontinued (Figure 2). Note that throughout the paper, the numbers of visits are shown in thousands, whereas expenses and revenue are shown in millions (US $).

To test the common trends assumption statistically, we also interacted pretreatment values with corresponding time dummies within the DID model (Figure 1). None of the coefficients were significant, suggesting that the trends are sufficiently common.

Again, to test the common trends assumption statistically, we also interacted pretreatment values with corresponding time dummies within the DID model (Figure 2). None of the coefficients were significant, suggesting that the trends are sufficiently common.

### Estimations

The estimation results are reported below in Table 3 (for adoption) and Table 4 (for discontinuation; model analyses were conducted with R [The R Foundation for Statistical Computing]). For brevity, control variables are not included in the tables, but they were included in all regressions along with hospital and time fixed effects. The interaction terms represent the DID effect, which represents the expected value of the additional difference between the treatment and control groups after treatment (ie, the end of the time trend), when first accounting for the differences in time trends and difference in treatment and control groups.

For *telehealth adoption*, we found the DID interaction term for total ambulatory visits to be positive and significant (*P*=.008). This means that the expected value of total ambulatory visits was higher in the treatment group than in the control group, even after accounting for the time and group differences, as well as several covariates discussed earlier and also in Multimedia Appendix 1. The average total ambulatory visits, as reported earlier, was 196 (thousand; SD 209 [thousand]). Given that the DID coefficient is 24.53 (thousand), this effect represents a significant increase in total ambulatory visits. Thus, we conclude that telehealth adoption resulted in more ambulatory visits for the adopting US hospitals during the period studied.

We further found the DID interaction term to be positive (*P*<.001) for the effect on total facility expenses. Thus, the expected value of total facility expenses was higher in the treatment group (ie, those that adopted telehealth) than in the control group (ie, similar hospitals that did not have, and did not adopt, telehealth during the same period). The average total facility expenses in our sample, as reported earlier, was (in millions) US $321.61 (SD US $342.3). The coefficient (in millions) is US $33.39 (*P*<.001), which represents a substantial average increase in the expenses when telehealth was adopted.

We also found the DID interaction term to be positive (*P*=.004) for the effect on hospital revenue, which suggests higher total revenue on average for those in the treatment group. The average total hospital revenue in our sample, as reported earlier, was (in millions) US $152.34. The coefficient (in millions) is $32.60 (*P*=.004), which represents a substantial average increase in the revenue when telehealth was adopted. However, we also note that this coefficient is slightly lower than that of the average increase in total facility expenses, suggesting that profits are likely to be negative or minimal when telehealth is first adopted.

The impact on ED visits (*P*=.36) was nonsignificant. The impact on outpatient services revenue was marginally significant (*P*=.01) and negative, suggesting that adoption led to at least a temporary drop in revenue, on average, in comparison with the control group.

For *telehealth discontinuation*, we found the DID interaction term (trt×post) to be significant and negative (*P*=.02) for the effect on outpatient services revenue. This means that the expected value for outpatient services revenue, ceteris paribus, was lower in the treatment group (ie, the group that discontinued telehealth) than in the control group, after accounting for the time trend and the assumed trend for the counterfactual. We also note that many control variables and fixed effects, to account for an unobserved time invariant heterogeneity, were accounted for. The average outpatient service revenue in our sample, as reported earlier, was (in millions) US $247.07. The coefficient (millions) is –US $65.37 (*P*=.02), which represents a substantial average drop in revenue compared with the control group when telehealth was discontinued.

We also found the DID interaction term to be negative and marginally significant (*P*=.09) for the effect on hospital revenue. This again means that the expected value, given all the aforementioned trends and variables, was lower in the treatment group than in the control group after treatment. The average hospital revenue for our sample, as reported earlier, was (in millions) US $188.32. Given that the coefficient (in millions) is –US $13.22, this represents a substantial potential average drop in total hospital revenue when telehealth was discontinued.

The impacts on ED visits (*P*=.10), total ambulatory visits (*P*=.28), and total facility expenses (*P*=.35) were nonsignificant.

**Table 3:** Difference-in-differences results for telehealth adoption.

	Emergency department visits (in thousands)^a^	*P* value	Total ambulatory visits (in thousands)^a^	*P* value	Outpatient services revenue (in millions, US $)^a^	*P* value	Total facility expenses (in millions, US $)^a^	*P* value	Hospital revenue (in millions, US $)^a^	*P* value
trt^b^	88.19 (6.19^c^)	<.001	64.10 (10.93)	<.001	–159.01 (19.86)	<.001	360.58 (5.49)	<.001	59.45 (24.76)	.02
post^d^	1.00 (1.52)	.51	–15.18 (6.96)	.03	29.85 (19.39)	.13	–16.29 (7.78)	.04	6.68 (21.85)	.77
trt×post	–1.49 (1.63)	.36	24.53 (9.15)	.008	–34.64 (20.71)	.10	33.39 (6.36)	<.001	32.60 (11.24)	.004
Hospital fixed effects	✓	N/A^e^	✓	N/A	✓	N/A	✓	N/A	✓	N/A
Time fixed effects	✓	N/A	✓	N/A	✓	N/A	✓	N/A	✓	N/A
(Intercept)	29.94 (2.63)	<.001	74.04 (5.60)	<.001	153.46 (16.46)	<.001	108.13 (3.80)	<.001	62.73 (23.61)	.008
n	499^f^	N/A	510	N/A	502^g^	N/A	510	N/A	502^h^	N/A
*R* ^2^	0.96	N/A	0.97	N/A	0.90	N/A	0.99	N/A	0.95	N/A
*F*-statistic	44.46	<.001	53.56	<.001	16.32	<.001	313	<.001	36.6	<.001

^a^All the dependent variables are Winsorized at 0.01 and 0.99 level.

^b^trt: treatment group.

^c^Robust SEs clustered at the hospital level (in parentheses).

^d^post: posttreatment time periods.

^e^N/A: not applicable.

^f^A total of 11 observations that did not have a mention of an emergency department visit were omitted from the model for emergency department visits, which is why the n is 499 instead of 510.

^g^A total of 8 observations that did not have a mention of outpatient services revenue were omitted from the model for outpatient services revenue, which is why the n is 502 instead of 510.

^h^A total of 8 observations that did not have a mention of hospital revenue were omitted from the model for hospital revenue, which is why the n is 502 instead of 510.

**Table 4:** Difference-in-differences results for telehealth discontinuation.

	Emergency department visits (in thousands)^a^	*P* value	Total ambulatory visits (in thousands)^a^	*P* value	Outpatient services revenue (in millions, US $)^a^	*P* value	Total facility expenses (in millions, US $)^a^	*P* value	Hospital revenue (in millions, US $)^a^	*P* value
trt^b^	26.86 (2.06^c^)	<.001	26.72 (4.98)	<.001	170.08 (37.33)	<.001	58.12 (12.30)	<.001	–104.41 (18.51)	<.001
post^d^	–0.25 (1.11)	.83	2.68 (6.23)	.67	–10.91 (29.27)	.71	0.07 (5.06)	.99	9.85 (10.40)	.34
trt×post	–1.65 (1.01)	.11	–7.84 (7.30)	.28	–65.37 (26.76)	.02	7.15 (7.65)	.35	–13.22 (7.70)	.09
Hospital fixed effects	✓	N/A^e^	✓	N/A	✓	N/A	✓	N/A	✓	N/A
Time fixed effects	✓	N/A	✓	N/A	✓	N/A	✓	N/A	✓	N/A
(Intercept)	32.49 (0.42)	<.001	29.91 (2.74)	<.001	22.70 (22.99)	.32	123.88 (8.24)	<.001	252.04 (11.35)	<.001
n	551^f^	N/A	552	N/A	552	N/A	552	N/A	549^g^	N/A
*R* ^2^	0.98	N/A	0.99	N/A	0.76	N/A	0.99	N/A	0.98	N/A
*F*-statistic	94.6	<.001	154.3	<.001	6.10	<.001	517.5	<.001	110.8	<.001

^a^All the dependent variables are Winsorized at 0.01 and 0.99 level.

^b^trt: treatment group.

^c^Robust SEs clustered at the hospital level (in parentheses).

^d^post: posttreatment time periods.

^e^N/A: not applicable.

^f^An observation that did not have a mention of an emergency department visit was omitted from the model for emergency department visits, which is why the n is 551 instead of 552.

^g^A total of 3 observations that did not have a mention of hospital revenue were omitted from the model for hospital revenue, which is why the n is 549 instead of 552.

### Robustness Checks

We conducted additional tests to address potential endogeneity issues and threats to validity. First, hospital management, not some central regulatory authority, makes telehealth adoption and discontinuation decisions. Thus, our sample has a potential self-selection endogeneity issue. To address this statistically, beyond the use of propensity score matching, we used a Heckman model [38,39]. The Heckman model consists of 2 stages and is designed to control for those omitted from the sample. The first stage models the self-selection decision, that is, whether a hospital adopts or discontinues telehealth. The second stage models the treatment effect while taking into consideration the selection decision by including the inverse mills ratio calculated from the first stage. The results of this robustness check are available in Multimedia Appendix 1 and are consistent with our primary results.

To test whether the outcomes were different for different years *after* treatment, we conducted 2-sample *t* tests using the 2018 data for group 1 (2 years after the treatment) versus the 2018 data of group 2 (1 year after the treatment). Recall that both group 1 and group 2 consist of treatment hospitals. Hospitals in group 1 are those that did not receive treatment in 2016, then received treatment in 2017 and 2018. Hospitals in group 2 are those that did not receive treatment in 2016 and 2017, then received treatment in 2018. The results are reported in Table 5 (for adoption) and Table 6 (for discontinuation).

We observed that after telehealth was adopted, there was an upward trend for the number of visits, expenses, and revenue when comparing year 2 to year 1 after the treatment (Table 5), although none are significant.

We observed that after telehealth was discontinued, there was no significant difference for most of the outcome variables, except for ED visits and total facility expenses (Table 6). For ED visits, we observed that the number of ED visits decreased further 2 years after the treatment compared with the previous year. The same trend of a further decrease 2 years after the treatment was found for total facility expenses.

**Table 5:** Results of comparison of outcome variables after treatment (year 1 vs year 2) after telehealth was adopted.

	Emergency department visits (in thousands)	Total ambulatory visits (in thousands)	Total facility expenses (in millions, US $)	Outpatient services revenue (in millions, US $)	Hospital revenue (in millions, US $)
Year 1, n, mean (SD); median	14, 46.03 (24.08); 44.61	14, 184.86 (163.96); 161.47	14, 329.75 (436.80); 193.85	14, 196.18 (88.89); 91.62	14, 137.33 (209.57); 42.19
Year 2, n, mean (SD); median	71, 55.15 (29.52); 47.47	71, 227.88 (256.83); 138.87	71, 389.40 (389.39); 323.32	71, 222.58 (407.19); 119.02	71, 213.31 (366.04); 96.29
*t* test, difference (SE)	9.12 (7.34)	43.02 (53.37)	59.66 (125.55)	26.40 (102.30)	75.99 (71.67)

**Table 6:** Results of comparison of outcome variables after treatment (year 1 vs year 2) after telehealth was discontinued.

	Emergency department visits (in thousands)	Total ambulatory visits (in thousands)	Total facility expenses (in millions, US $)	Outpatient services revenue (in millions, US $)	Hospital revenue (in millions, US $)
Year 1, n, mean (SD); median	80, 55.01 (35.37); 48.72	80, 243.16 (341.72); 111.67	80, 456.60 (589.83); 247.90	80, 172.74 (224.79); 91.62	80, 193.38 (333.23); 77.20
Year 2, n, mean (SD); median	12, 42.72 (18.65); 39.10	12, 179.07 (134.64); 134.65	12, 239.61 (155.71); 199.68	12, 150.55 (103.77); 162.36	12, 95.81 (78.31); 75.31
*t* test, difference (SE), *P* value	–12.29 (6.68), .03	–64.09 (54.61), .12	–216.98 (79.80), .003	–22.19 (38.47), .28	–97.57 (43.58), .01

## Discussion

### Overview

This study assessed the impact of telehealth video visit consultation adoption or discontinuation by US hospitals from 2016 to 2018 through analysis of 2 quasi-natural experiments (ie, one for adoption and one for discontinuation). After conducting a number of robustness checks to validate our findings, we can conclude that, for this period, telehealth adoption resulted in an average increase in total ambulatory visits, total facility expenses, and hospital revenue in comparison with the control group of similar hospitals that neither offered, nor had adopted, telehealth services during this same period. Telehealth discontinuation resulted in an average reduction in outpatient services revenue compared with the control group of similar hospitals that did not discontinue telehealth during this period. Furthermore, in our robustness check, we found telehealth discontinuation to reduce total facility expenses over time, suggesting that telehealth investments are costly and cannot simply rely on existing communications infrastructure (ie, it is not the case that little to no additional costs are involved).

### Principal Findings

First, we found that telehealth adoption for US hospitals from 2016 to 2018 resulted in increased visits, expenses, and revenue in comparison with the control group. These findings are similar to those of another study that found telehealth not only increased use (ie, resulted in more visits), but also increased costs [3]. However, this previous study focused on direct-to-consumer telehealth for a payer-based patient population in California as opposed to telehealth offered by hospitals throughout the United States. Thus, we contribute by demonstrating a similar trend at the national level and for hospital-based (provider-based) telehealth as opposed to payer-supported direct-to-consumer telehealth. The implications of our findings are that providers switching from not offering telehealth to offering telehealth can expect higher visit volumes but not necessarily significant increases in profits, especially given that the coefficient for increased expenses (US $33.39 million) is slightly higher than the coefficient for increased revenue (US $32.60 million) in our telehealth adoption results. The results make sense because it has been found that offering telehealth can increase provider workload [40], reduce workflow efficiency (at first) [23,41], and result in billing and payment issues [42]. Furthermore, given that payment parity laws are only now becoming more commonplace for telehealth and are still subject to significant variability [43], revenue from additional telehealth visits may be less than expected, especially if visits that were typically in person are now being replaced with video-based visits. Thus, telehealth adoption may provide more convenience for patients but may have mixed impacts on provider performance, likely requiring a significant investment by providers in overcoming barriers at least in the short term, as was also found in other telehealth studies such as those in the area of telestroke [44].

Second, we found that telehealth discontinuation had a negative impact on outpatient services revenue. The implication is that once telehealth is offered, performance may subsequently suffer if it is discontinued. Thus, careful thought must be given to what might happen with patient expectations once telehealth is offered, even if only for a short time. However, we also note that, although the observed decline in visit volume might be expected to be responsible for loss in revenue, we did not find a significant impact on total ambulatory visits in comparison with the control group when telehealth was discontinued. This means that the revenue loss may be attributable to a decline in other outpatient services such as wellness and prevention programs, observation programs, supplies, laboratory tests, or other services, which suggests a spillover effect. Future research could examine this effect in more detail to gain a deeper understanding of potential spillover effects between discontinuing a digital service and other outpatient services offered. Most importantly, spillover effects aside, our results demonstrate that offering a digital service may change expectations, which cannot simply be reverted if telehealth is then no longer offered in a future period.

### Limitations

We note that this study is limited by the binary nature of the response variable in that telehealth is a yes or no variable rather than an extent of use or assimilation variable. We also note that our data dates to before the COVID-19 pandemic period in which telehealth adoption and use significantly increased at first but subsequently significantly declined [8,45]. Future research could consider whether the effects found in this study are consistent with the postpandemic period, once more data are available. This study is also limited by a lack of detail in regard to the mechanisms that cause the effects we observed. This is also a significant opportunity for future research. Finally, our data are limited to the United States.

### Additional Thoughts on Future Research

In addition to studying the spillover effects of telehealth adoption and discontinuation decisions, as well as determination of whether the effects found here remain consistent after the pandemic once more data are available, future research could consider price optimization for service channel differences such as in-person versus video visits and establish recommendations for optimal mixes of visit types, conditional on patient conditions and provider expertise. Given that the relationship among telehealth use, costs, and revenue is complex, uncertain, and mixed, more research is needed on service mix optimization.

We further note that our results are specific to US hospitals. Future research could consider whether these results are consistent with telehealth being adopted and discontinued in other countries and regions, as well as any unique conditions that may affect telehealth differently in other areas.

Finally, telehealth impacts, especially from adoption of telehealth, are likely to change over time. For instance, costs associated with telehealth may decrease in some ways as efficiencies are gained over time but increase in other ways such as potentially more technical and scheduling staff being required to support a mix of in-person and telehealth visits. Therefore, an excellent future area for future research will be a more fine-grained analysis of telehealth-specific costs over a longer period of time.

### Conclusions

In conclusion, this study offers insights into the effects of telehealth adoption and discontinuation by US hospitals from 2016 to 2018. It is our hope that these results will inform health care providers, administrators, and policy makers regarding expected performance outcomes when telehealth adoption and discontinuation decisions are made.

## Appendix

Multimedia Appendix 1 Additional descriptions, first stage results, and robustness checks.

## Abbreviations

ADI: Area Deprivation Index
AHA: American Hospital Association
DID: difference-in-differences
ED: emergency department
HCRIS: Healthcare Cost Report Information System
