# Supplementary material for: Telehealth Adoption and Discontinuation by US Hospitals: Results From 2 Quasi-Natural Experiments
Source: JMIR Form Res. 2022 Feb 18;6(2):e28979. doi: 10.2196/28979 (PMC8900896; doi:10.2196/28979)
Supplement: Multimedia Appendix 1 [file formative_v6i2e28979_app1.docx]

# Telehealth Adoption and Discontinuation by U.S. Hospitals: Results from Two Quasi-Natural Experiments

## SUPPLEMENTARY APPENDIX

## Outcome Variable Descriptions

The following information was aggregated from the CMS HCRIS Worksheet explanations and the AHA Annual Survey Instructions and Definitions:

**ED Visits:** Total facility emergency department visits. Should reflect the number of visits to the emergency unit. Emergency outpatients can be admitted to the inpatient areas of the hospital, but they are still counted as emergency visits and subsequently as inpatient admissions.

**Total Ambulatory Visits (minus ED visits):** A visit by a patient who is not lodged in the hospital while receiving medical, dental, or other services. Each appearance of an outpatient in each unit constitutes one visit regardless of the number of diagnostic and/or therapeutic treatments that the patient receives. Total outpatient visits should include all clinic visits, referred visits, observation services, outpatient surgeries (also reported on line E1k), home health service visits, and emergency department visits (also reported on line E1g). (We note that we used the field in the AHA data that included all outpatient visits minus ED visits, so as not to overlap with the ED visits variable.) Clinic visits should reflect total number of visits to each specialized medical unit that is responsible for the diagnosis and treatment of patients on an outpatient, nonemergency basis. (e.g., alcoholism, dental, gynecology.) Visits to the satellite clinics and primary group practices should be included if revenue is received by the hospital.

**Outpatient Services Revenue:** Revenue from outpatient services. (Further information: (<https://www.costreportdata.com/instructions/Instr_G200.pdf>, <https://www.cms.gov/Regulations-and-Guidance/Guidance/Manuals/downloads/bp102c06.pdf>, https://www.medicare.gov/coverage/outpatient-hospital-services)

**Total Facility Expenses:** Includes all payroll and non-payroll expenses as well as any nonoperating losses (including extraordinary losses and bad debt). Sum of payroll expenses, employee benefits, depreciation expense (for reporting period only), interest expense, pharmacy expense, supply expense (other than pharmacy), and all other expenses.

**Hospital Revenue:** Includes all revenue from in-patient services, ancillary services, outpatient services, rural health care, home health, ambulance services, rehabilitation, ambulatory surgery, hospice, and any other service revenue a hospital reports in the “Other” category.

## Additional Results and Details

**Table A1a: Logistic regression results for propensity scoring for telehealth adoption**

| **Variable** | **Estimate (Std. Error)** |
| --- | --- |
| SystemOwned | 2.190 *** (0.311) |
| WageIndx | -0.895 (1.678) |
| HITAssetCost | -23128.4 (24747.954) |
| TotalAdmAndVisits | 0.665 (0.615) |
| HHI | -1.592 (1.629) |
| PercMdcdElig | -3.082 (3.278) |
| COTH | 0.751 (0.524) |
| Own_FP | -0.291 (0.675) |
| Own_NP | 0.797 + (0.468) |
| PercCapit | 0.012 (0.025) |
| PercRsk | 0.081 (0.034) |
| Region_MW | 0.096 (0.523) |
| Region_S | 0.086 (0.617) |
| Region_W | 0.692 (1.098) |
| CMI | -0.504 (0.485) |
| CntyHlthRank | 0.469 (0.857) |
| CntyPercPop65 | -17.659 ** (6.244) |
| CntyPercBlack | -0.006 (0.017) |
| CntyPercNative | 0.419 (0.300) |
| CntyPercLatino | -0.054 ** (0.017) |
| CntyPercDeepPoverty | -0.346 *** (0.102) |
| CntyPercDisabled | -0.042 (0.103) |
| CntyBBMaxUp | 0.008 (0.006) |
| CntyHsholdIntUse | -9.028 (6.814) |
| CntyADI | 0.352 + (0.193) |
| Year | -0.201 (0.175) |
| (Intercept) | 16.214 * (7.689) |
| n | 469 |
| Pseudo-R-squared | 0.28 |
| Wald chi-squared | 153.51*** |
| Notes: DV is hospital in treatment group = 1; Own_Gov and Region_NE are omitted; Robust standard errors are in parentheses and are clustered by hospital. | |

**Table A1b: Logistic regression results for propensity scoring for telehealth discontinuation**

| **Variable** | **Estimate (Std. Error)** |
| --- | --- |
| SystemOwned | -0.759*** (0.197) |
| WageIndx | 0.922 (0.840) |
| HITAssetCost | 0.000 (0.000) |
| TotalAdmAndVisits | 0.000 (0.000) |
| HHI | -0.889 (0.609) |
| PercMdcdElig | 4.887** (1.722) |
| COTH | 0.827*** (0.221) |
| Own_FP | 0.627 (0.426) |
| Own_NP | 1.203*** (0.296) |
| PercCapit | 0.002 (0.024) |
| PercRsk | -0.009 (0.011) |
| Region_MW | -0.291 (0.274) |
| Region_S | 0.963** (0.303) |
| Region_W | -0.732 (0.830) |
| CMI | -1.787*** (0.357) |
| CntyHlthRank | 1.578*** (0.480) |
| CntyPercPop65 | -3.120 (2.613) |
| CntyPercBlack | -0.021* (0.008) |
| CntyPercNative | -0.045 (0.032) |
| CntyPercLatino | 0.001 (0.009) |
| CntyPercDeepPoverty | 0.012 (0.050) |
| CntyPercDisabled | -0.136** (0.051) |
| CntyBBMaxUp | -0.003 (0.003) |
| CntyHsholdIntUse | 0.784 (2.919) |
| CntyADI | -0.253* (0.104) |
| Year | 0.102 (0.094) |
| (Intercept) | -0.724 (3.472) |
| n | 1545 |
| Pseudo-R-squared | 0.09 |
| Wald chi-squared | 135.05*** |
| Notes: DV is hospital in treatment group = 1; Own_Gov and Region_NE are omitted; Robust standard errors are in parentheses and are clustered by hospital. | |

**Table A2a: Probit regression results for Heckman first stage model for adoption.**

| **Variable** | **Estimate (Std. Error)** |
| --- | --- |
| SystemOwned | 1.282 *** (0.176) |
| WageIndx | -0.499 (0.954) |
| HITAssetCost | -0.012 (0.014) |
| TotalAdmAndVisits | 0.000 (0.000) |
| HHI | -0.975 (0.919) |
| PercMdcdElig | -2.052 (1.836) |
| COTH | 0.412 (0.295) |
| Own_FP | -0.204 (0.388) |
| Own_NP | 0.483 + (0.266) |
| PercCapit | 0.007 (0.013) |
| PercRsk | 0.043 * (0.0178) |
| Region_MW | 0.0704 (0.294) |
| Region_S | 0.024 (0.348) |
| Region_W | 0.391 (0.644) |
| CMI | -0.223 (0.276) |
| CntyHlthRank | 0.264 (0.485) |
| CntyPercPop65 | -9.844 *** (3.609) |
| CntyPercBlack | -0.003 (0.010) |
| CntyPercNative | 0.251 (0.167) |
| CntyPercLatino | -0.032 *** (0.010) |
| CntyPercDeepPoverty | -0.199 *** (0.056) |
| CntyPercDisabled | -0.032 (0.058) |
| CntyBBMaxUp | 0.004 (0.003) |
| CntyHsholdIntUse | -5.603 (3.863) |
| CntyADI | 0.218 * (0.108) |
| Year | -0.119 (0.100) |
| (Intercept) | 9.630 * (4.371) |
| n | 1545 |
| Pseudo-R-squared | 0.09 |
| Wald chi-squared | 132.38*** |
| Notes: DV is hospital in treatment group = 1; Own_Gov and Region_NE are omitted; Robust standard errors are in parentheses and are clustered by hospital. | |

**Table A2a: Probit regression results for Heckman first stage model for discontinuation**

| **Variable** | **Estimate (Std. Error)** |
| --- | --- |
| SystemOwned | -0.458*** (0.113) |
| WageIndx | 0.540 (0.469) |
| HITAssetCost | 0.000 (0.000) |
| TotalAdmAndVisits | 0.000 (0.000) |
| HHI | -0.481 (0.338) |
| PercMdcdElig | 2.863** (1.001) |
| COTH | 0.458*** (0.126) |
| Own_FP | 0.434+ (0.235) |
| Own_NP | 0.686*** (0.160) |
| PercCapit | 0.001 (0.013) |
| PercRsk | -0.005 (0.006) |
| Region_MW | 0.146 (0.153) |
| Region_S | 0.520** (0.170) |
| Region_W | -0.252 (0.410) |
| CMI | -0.989*** (0.196) |
| CntyHlthRank | 0.833*** (0.270) |
| CntyPercPop65 | -1.857 (1.459) |
| CntyPercBlack | -0.011* (0.005) |
| CntyPercNative | -0.027 (0.016) |
| CntyPercLatino | 0.001 (0.005) |
| CntyPercDeepPoverty | 0.002 (0.028) |
| CntyPercDisabled | -0.076** (0.028) |
| CntyBBMaxUp | -0.002 (0.002) |
| CntyHsholdIntUse | 0.374 (1.646) |
| CntyADI | -0.130* (0.059) |
| Year | 0.058 (0.053) |
| (Intercept) | -0.465 (1.962) |
| n | 1545 |
| Pseudo-R-squared | 0.09 |
| Wald chi-squared | 132.38*** |
| Notes: DV is hospital in treatment group = 1; Own_Gov and Region_NE are omitted; Robust standard errors are in parentheses and are clustered by hospital. | |

**Table A3a: Heckman Difference-in-Differences Results for telehealth adoption**

|  | **EDVisits**  **(in thousands)** | **TotalAmbVisits**  **(in thousands)** | **TotFacExp**  **(in millions)** | **OutpatSerRev**  **(in millions)** | **HospRev**  **(in millions)** | | |
| --- | --- | --- | --- | --- | --- | --- | --- |
| trt | 83.26 (8.67) *** | 194.99 (111.10) + | 300.20 (61.01) *** | -168.04 (176.44) | 11.52 (70.61) | | |
| post | 1.28 (1.57) | -14.36 (7.76) | -15.20 (8.35) * | 32.58 (20.62) | 14.24 (22.77) | | |
| trt*post | -1.43 (1.81) | 18.92 (8.09) * | 32.59 (6.86) *** | -44.04 (22.76) + | 23.55 (8.77) ** | | |
| Inv. Mills Ratio | 0.69 (2.29) | -33.43 (30.17) | 16.20 (16.20) | 3.65 (46.27) | 9.57 (19.00) | | |
| Hosp. Fixed Effects | √ | √ | √ | √ | √ | | |
| Time Fixed Effects | √ | √ | √ | √ | √ | | |
| (Intercept) | 31.13 (5.09) *** | 9.21 (59.49) | 141.05 (33.40) *** | 161.78 (97.76) + | 83.79 (41.19) * | | |
| n | 499 | 510 | 510 | 502 | 502 | | |
| R^2^ | 0.97 | 0.97 | 0.99 | 0.87 | 0.98 | | |
| F-Stat | 47.1*** | 49.1*** | 299.8*** | 10.84*** | 110.8 *** | | |
| Notes: +p<0.10, * p<0.05, ** p<0.01, *** p<0.001; Robust standard errors reported in parentheses. | | | | | |  |  |

**Table A3b: Heckman Difference-in-Differences Results for telehealth discontinuation**

|  | **EDVisits**  **(in thousands)** | **TotalAmbVisits**  **(in thousands)** | **TotFacExp**  **(in millions)** | **OutpatSerRev**  **(in millions)** | **HospRev**  **(in millions)** | | |
| --- | --- | --- | --- | --- | --- | --- | --- |
| trt | 27.99 (7.55) *** | -167.59 (64.06) ** | -25.37 (93.65) | 1013.41 (640.15) | -134.49 (97.19) | | |
| post | -0.77 (0.99) | 0.56 (5.50) | -0.39 (5.75) | -1.61 (27.46) | 11.71 (10.79) | | |
| trt*post | -1.62 (1.03) | -7.49 (7.70) | 8.18 (8.22) | -62.09 (26.66) * | -13.21 (8.08) | | |
| Inv. Mills Ratio | -0.56 (4.10) | 111.03 (37.36) ** | 47.29 (52.96) | -483.77 (372.74) | 16.79 (55.02) | | |
| Hosp. Fixed Effects | √ | √ | √ | √ | √ | | |
| Time Fixed Effects | √ | √ | √ | √ | √ | | |
| (Intercept) | 32.24 (2.41) *** | 91.30 (21.42) *** | 149.92 (28.69) *** | -246.61 (204.79) | 261.59 (32.34) *** | | |
| n | 537 | 538 | 538 | 538 | 538 | | |
| R^2^ | 0.98 | 0.98 | 0.99 | 0.74 | 0.98 | | |
| F-Stat | 103.3*** | 155.8*** | 497.9*** | 5.33*** | 110.8 *** | | |
| Notes: +p<0.10, * p<0.05, ** p<0.01, *** p<0.001; Robust standard errors reported in parentheses. | | | | | |  |  |

We note that the coefficient directions and significance levels are consistent with the DID results reported earlier. We also note, though, that the inverse mills ratio (IMR) is only significant (p<0.01) for the TotalAmbVisits model. This mixture of significance of the IMR for this model and non-significance for the other models raises the question of whether or not selection correction is required in this case. Opting for a conservative and thorough approach, we report results from these models.

**Table A4a: DID results using one-to-one matching for telehealth adoption**

|  | **EDVisits**  **(in thousands)** | **TotalAmbVisits**  **(in thousands)** | | **TotFacExp**  **(in millions)** | **OutpatSerRev**  **(in millions)** | | | **HospRev**  **(in millions)** | | |  |
| --- | --- | --- | --- | --- | --- | --- | --- | --- | --- | --- | --- |
| trt | 96.10 (8.56) *** | 104.69 (15.34) *** | 386.61 (5.15) *** | | -6.90 (19.57) | | 91.72 (17.57) *** | | | |  |
| post | 1.17 (1.82) | -13.21 (7.54) + | -17.22 (7.80) * | | 18.96 (19.44) | | 10.81 (22.26) | | | |  |
| trt*post | -1.17 (1.93) | 24.01 (9.25) ** | 33.58 (6.37) *** | | -25.18 (20.92) | 27.82 (11.13) * | | | | |  |
| Hosp. Fixed Effects | √ | √ | √ | | √ | √ | | | | |  |
| Time Fixed Effects | √ | √ | √ | | √ | √ | | | | |  |
| (Intercept) | 22.04 (6.42) *** | 33.72 (11.89) ** | 82.08 (3.24) *** | | 0.70 (17.54) | 30.74 (16.28) + | | | | |  |
| n | 495 | 510 | 510 | | 497 | 497 | | | | |  |
| R^2^ | 0.95 | 0.96 | 0.99 | | 0.92 | 0.95 | | | | |  |
| F-Stat | 36.97 *** | 52.88 *** | 315.4 *** | | 17.29 *** | 37.13 *** | | | | |  |
| Notes: +p<0.10, * p<0.05, ** p<0.01, *** p<0.001; Robust standard errors reported in parentheses. | | | | | | | | |  |  | |

**Table A4b: DID results using one-to-one matching for telehealth discontinuation**

|  | **EDVisits**  **(in thousands)** | **TotalAmbVisits**  **(in thousands)** | | **TotFacExp**  **(in millions)** | **OutpatSerRev**  **(in millions)** | | | **HospRev**  **(in millions)** | | |  |
| --- | --- | --- | --- | --- | --- | --- | --- | --- | --- | --- | --- |
| trt | 18.26 (3.02) *** | 53.01 (6.07) *** | 118.66 (11.56) *** | | 122.27 (38.86) ** | | 105.69 (17.57) *** | | | |  |
| post | -0.22 (1.93) | -7.96 (10.99) | 4.09 (7.73) | | -4.21 (32.99) | | 14.37 (8.07) + | | | |  |
| trt*post | -2.07 (1.69) | -2.13 (9.85) | 2.52 (9.62) | | -80.77 (32.06) * | -24.19 (8.80) * | | | | |  |
| Hosp. Fixed Effects | √ | √ | √ | | √ | √ | | | | |  |
| Time Fixed Effects | √ | √ | √ | | √ | √ | | | | |  |
| (Intercept) | 41.59 (2.03) *** | 4.46 (3.43) | 63.11 (6.80) *** | | 73.84 (23.76) ** | 44.03 (8.76) *** | | | | |  |
| n | 551 | 552 | 552 | | 552 | 542 | | | | |  |
| R^2^ | 0.98 | 0.98 | 0.99 | | 0.84 | 0.98 | | | | |  |
| F-Stat | 79.99 *** | 84.22 *** | 386.3 *** | | 10.23 *** | 116.8 *** | | | | |  |
| Notes: +p<0.10, * p<0.05, ** p<0.01, *** p<0.001; Robust standard errors reported in parentheses. | | | | | | | | |  |  | |

**Table A5a: Heckman DID results using one-to-one matching for telehealth adoption**

|  | **EDVisits**  **(in thousands)** | **TotalAmbVisits**  **(in thousands)** | **TotFacExp**  **(in millions)** | **OutpatSerRev**  **(in millions)** | **HospRev**  **(in millions)** | | |
| --- | --- | --- | --- | --- | --- | --- | --- |
| trt | 109.53 (13.14) *** | 299.33 (159.77) + | 287.40 (88.06) ** | 192.53 (242.36) | -45.07 (89.74) | | |
| post | 1.20 (2.04) | -12.54 (8.73) | -16.72 (8.77) + | 23.33 (21.60) | 16.62 (23.73) | | |
| trt*post | -1.75 (2.37) | 17.71 (8.12) * | 33.98 (7.40) *** | -35.53 (24.23) | 21.07 (9.14) * | | |
| Inverse Mills Ratio | -2.84 (2.49) | -48.82 (39.57) | 24.31 (21.13) | -49.37 (57.73) | 30.48 (20.31) | | |
| Hosp. Fixed Effects | √ | √ | √ | √ | √ | | |
| Time Fixed Effects | √ | √ | √ | √ | √ | | |
| (Intercept) | 10.91 (10.13) | -68.76 (92.38) | 139.97 (51.97) ** | -107.97 (143.90) | 104.56 (56.06) + | | |
| n | 495 | 510 | 510 | 497 | 497 | | |
| R^2^ | 0.96 | 0.97 | 0.99 | 0.88 | 0.97 | | |
| F-Stat | 34.0 *** | 44.92 *** | 281.1*** | 11.48 *** | 47.52 *** | | |
| Notes: +p<0.10, * p<0.05, ** p<0.01, *** p<0.001; Robust standard errors reported in parentheses. | | | | | |  |  |

**Table A5b: Heckman DID results using one-to-one matching for telehealth discontinuation**

|  | **EDVisits**  **(in thousands)** | **TotalAmbVisits**  **(in thousands)** | **TotFacExp**  **(in millions)** | **OutpatSerRev**  **(in millions)** | **HospRev**  **(in millions)** | | |
| --- | --- | --- | --- | --- | --- | --- | --- |
| trt | 17.69 (7.58) * | -128.66 (61.36) * | 6.80 (84.03) | 990.65 (602.94) | 6.97 (75.05) | | |
| post | 0.56 (1.25) | -7.04 (8.89) | 2.86 (8.48) | 7.34 (36.51) | 15.70 (9.17) + | | |
| trt*post | -2.28 (1.38) | -1.27 (10.94) | 0.55 (10.21) | -84.74 (34.89) * | -26.81 (9.93) ** | | |
| Inverse Mills Ratio | 0.26 (4.04) | 104.12 (36.08) ** | 52.39 (48.78) | -500.81 (342.44) | 57.60 (41.87) | | |
| Hosp. Fixed Effects | √ | √ | √ | √ | √ | | |
| Time Fixed Effects | √ | √ | √ | √ | √ | | |
| (Intercept) | 41.63 (2.92) *** | 60.41 (20.14) ** | 97.39 (26.49) *** | -198.73 (189.28) | 73.94 (24.88) ** | | |
| n | 551 | 552 | 552 | 542 | 542 | | |
| R^2^ | 0.98 | 0.98 | 0.99 | 0.85 | 0.98 | | |
| F-Stat | 109.4 *** | 81.52 *** | 372.8 *** | 9.68 *** | 106.6 *** | | |
| Notes: +p<0.10, * p<0.05, ** p<0.01, *** p<0.001; Robust standard errors reported in parentheses. | | | | | |  |  |
